# PEDF/PEDF-R Signaling Regulates Retinal Phospholipid Homeostasis and Photoreceptor Survival

**DOI:** 10.64898/2026.08.04.742541

**Published:** 2026-08-09

**Authors:** Alexandra Bernardo-Colón, Susan E Crawford, Martin Paul Agbaga, Zhen Wang, Kevin Schey, S. Patricia Becerra

## Abstract

Pigment epithelium-derived factor (PEDF) promotes photoreceptor survival through its receptor PEDF-R, a phospholipase involved in retinal lipid metabolism. To define the in vivo function of the PEDF/PEDF-R axis, we generated mice lacking *Serpinf1* (PEDF) and *Pnpla2* (PEDF-R). Combined loss of *Serpinf1* and *Pnpla2* resulted in severe retinal degeneration characterized by outer nuclear layer (ONL) thinning, outer segment (OS) shortening, reduced rhodopsin and cone opsin expression, increased TUNEL-positive nuclei, and enhanced retinal autofluorescence associated with altered lipid distribution. Lipid-associated markers, including TIP47, PLIN5, and BODIPY, exhibited abnormal distribution patterns in mutant retinas, indicating disrupted lipid storage and trafficking. Loss of PEDF/PEDF-R signaling also impaired photoreceptor-rod bipolar cell connectivity, as demonstrated by reduced PKCα/synaptophysin colocalization, and resulted in diminished electroretinographic responses. Lipid Imaging mass spectrometry revealed decreases in some lipid abundances in photoreceptor outer segment and inner segment/outer nucleus layer, while lipids containing arachidonic acid and docosahexaenoic acid-containing lipids showed increased abundance. Together these findings identify the PEDF/PEDF-R signaling axis as a key regulator of retinal phospholipid homeostasis that couples lipid metabolism to photoreceptor survival and visual function.

## Full Text Availability

The license terms selected by the author(s) for this preprint version do not permit archiving in PMC. The full text is available from the preprint server.

